# Progress in State Medicine

**Published:** 1903-03-28

**Authors:** 


### Progress in State Medicine.

### TUBERCULOSIS.

Cameron[1] states that it is only within a recent period that tuberculosis has with certainty been classed with the contagious diseases. As regards pulmonary tuberculosis, it seems almost certain that the chief cause of this form of the malady is the inhalation of the dried expectorated matter from the lungs of affected persons. The offensive habit of expectorating on floors, passages, in trains, omnibuses, and railway carriages is largely the indirect cause of consumption. Some slight decrease of this objectionable habit has been noticed in some towns, owing to notices against it being put up in various places, and leaflets explaining its dangers issued by sanitary authorities and associations. Whilst precautions with regard to the sputum are taken in consumptives' hospitals and the phthisis wards of other hospitals, little is being done in this respect in the case of patients living at home. If the disease were notifiable the sanitary authority would be in a position to supply spittoons and disinfectants to the poorer patients. In Dublin a large number of cases are voluntarily notified. A poor patient discovered residing with his family or in lodgings is supplied with a pasteboard spittoon containing an absorbent material and a disinfectant, and with printed information relating to consumption. The spittoon is subsequently called for, brought to a destructor, and there it is, together with its contents, destroyed. Unfortunately, in many cases, the consumptives do not avail themselves of help or advice from the sanitary authority, and even when far advanced in the disease object to be regarded as consumptives. The compulsory notification of at least tuberculosis of the lungs is a factor absolutely necessary in the prevention of consumption. The proper thing would be to obtain an Act of Parliament enabling sanitary authorities to make lung tuberculosis compulsorily notifiable, but protecting the patients from such of the sections of the Public Health Acts as would unduly interfere with their movements. Voluntary notification has not given very satisfactory results where adopted. The disinfection of rooms vacated by consumptives is very generally neglected in the houses occupied by the poor. Every week he received from the Registrar-General of Ireland a list of the places in which deaths from lung tuberculosis have occurred. In about 40 per cent. of the places permission to disinfect them is given. Rohe has shown that wall scrapings from rooms occupied by consumptives inoculated into animals produced in 20 per cent. of them tuberculosis. Sanitary authorities should make provision for receiving the poor consumptives into sanatoria. Niven[2] points out that in no fewer than 19 out of the 44 counties the female death-rate from phthisis exceeds the male death-rate. In 30 out of the 33 great towns the male death-rate from phthisis exceeds the female, often considerably. In three towns—Gateshead, Sunderland, and Swansea—the female death-rate slightly exceeds the male. All three lie in the colliery districts, and it would almost seem as if coal mining acted as a protective against tuberculous phthisis. That intemperance has a decided effect in the production of phthisis is shown by the close association between its extent in particular occupations, and a high phthisis death-rate, as well as by the excessive mortality from phthisis amongst publicans and their servants. This is accounted for by the infection which depends on the general habit of spitting on the floor, and which assails as well the persons attending the customers as the customers themselves. The heaviest phthisical incidence will be found in those trades attended with the production of much dust. It may also be expected in very heavy and hot occupations, occupations which, besides their direct effects, tend to produce intemperance. In all these the lung is liable to injury, and to receive tuberculous infection from some quarter. The extent to which it is transmitted will be determined partly by the habit of spitting, partly by the prevalence of dust, which leads to the rapid drying of the sputum. In Manchester, the chief source of infection is the presence of another case at home. There is evidence from individual cases of the disease having been contracted by persons cleaning out public offices, by upholsterers, and persons cleaning out railway carriages, and by house decorators. A number of instances also occur in which the infection appears to have been contracted in public-houses. The principal measures to protect workpeople against this disease are (1) Compulsory notification of all cases of tuberculous phthisis, so that every operative may be instructed in the precautions needed to protect his fellow workmen;

(2) all spitting in workshops should be prohibited; (3) the systematic, daily, wet sweeping of workrooms. Workpeople engaged in dusty occupations should be taught to breathe through their nostrils. Public-houses should be placed under strict legislation in respect of cleanliness, and spitting in public-houses should be prohibited. Niven is satisfied that the public-house is a large manufactory of phthisis among workers, and should receive special attention. Hospital provision is needed at the expense of the rates for the removal of consumptives from crowded homes. Harris[3] considers the chief factors in the cause of phthisis are :—(1) Heredity; (2) soil; (3) occupation. The marriage of consumptive people cannot be prevented generally, although it is a folly that is almost criminal. The heaviest mortality from this disease is seen in the peat manufacturing centres in Lancashire, Cheshire, and Yorkshire. The inhalation of trade dust was a powerful factor in producing the disease, ranging from 69·6 per cent. among needle polishers to 7 per cent. among bakers. The hardest dusts produced the worst effects. Lord Inverclyde[4] states that there is a growing opinion that consumption ought to be dealt with by some modified method of isolation, because it was recognised on all hands that it was in some degree an infectious disease. Impure air must be treated as being as great a danger as impure water. Indiscriminate expectoration was not only very offensive, but was insanitary and dangerous. Sanatoria should be established throughout the country where poor persons could be treated while the disease was still in a state when it could be dealt with. Neech,[5] whilst agreeing with the provision of sanatoria for the cure of those who have already contracted phthisis, thinks that if this disease is to be combated with success, and the death-rate therefrom markedly diminished within a reasonable time, not only must curative means be provided, but preventive action must also be carried out in a most vigorous and thorough manner. Voluntary notification only touches the fringe of the matter. Notification to be of any use must be compulsory. Notification to the sanitary authority does not mean notification to the public. He states that Baker, Secretary to the State Board of Health of Michigan, where compulsory notification of phthisis has been in operation for several years, says: "That they do not find notification of consumption to cause any inconvenience to the patients." A consumptive who takes care to collect and destroy his sputum is not a danger to the public. Compulsory notification in New York has brought out and emphasised three important facts, viz. :—(1) That the disease occurs chiefly in infected areas. (2) That fresh cases crop up year after year in individual houses infected with the disease. (3) That compulsory notification followed by the sanitary precautions which such notification renders possible, leads to a marked diminution in the death-rate from the disease. In New York the death-rate has fallen 30 per cent. In all cases of phthisis notified he would advocate no interference with a patient, so long as such patient was under the care of a qualified medical man. Littlejohn[6] refers to the general prevalence of the disease, to its comparative mortality in this country and in Edinburgh, and to the great decline in the death-rate during the Victorian Era. The prevention of consumption and every other infectious disease was a duty of the municipality, with the co-operation of the medical profession. The main objections to notification were :—(1) The great uncertainty of diagnosis; (2) its chronic nature which prevented its being controlled like acute infectious diseases; (3) that it was not in all cases infectious by contact; (4) ordinary preventive measures were said not to be effective; (5) notification would lead to social ostracism and the consumptive would lose his work; (6) that the medical attendant would be interfered with. The advantages of notification were :—(1) A knowledge of the disease, its existence and behaviour; (2) knowledge of sanitary defects; (3) that it would permit of the education of the patient and his family. Hillier[7] thinks that the prospects of extinguishing tuberculosis in this country depend upon the enterprise and sense of duty which actuate that part of the public service known as public health. The English decline in the tuberculosis death-rate had been a fairly constant one. In England the deaths from phthisis had been declining since they stood at 38 per 10,000; they had now reached 13 per 10,000. The drop was associated with and largely attributed to the general improvement in sanitary conditions and food standards among the working classes. He states that Koch attributes this decline, both in England and in Germany, not so much to sanatoria as to the knowledge of the infectious character of the disease and the consequent precautions brought into practice. He suggests that a Bill be presented at the next session of Parliament embracing the following points :—(1) Compulsory notification of phthisis; (2) such a degree of isolation of all advanced cases as is afforded by sanatoria or special hospitals, wherever such isolation is necessary or desirable in the opinion of the medical officer of health; (3) a provision for an increase in the staffs of all medical officers of health, such increase to be specially devoted to the supervision and control of tuberculosis; and (4) authorisation to medical officers of health to appropriate small-pox hospital accommodation for cases of phthisis wherever in their opinion such accommodation exceeds the demand at all likely to be made by the occurrence of small-pox. It was perfectly reasonable to suppose that public health control of phthisis would eventually stamp out the disease. Dalton[8] points out in his report that not only is the amount of consumption in the borough of Cambridge smaller than in many other districts, but it has markedly decreased of late. There is evidence to show that many of the cases are the result of infection from other members of the family living in the same house. The problem therefore is not so much the provision of sanatoria for consumptives as of means to prevent the spread of the disease from one member of a family to another. He suggests the distribution of pamphlets to patients, and that visits be occasionally made to ascertain that the proper precautions are carried out. He further recommends that the sanitary authority be informed of any fresh cases and of deaths from phthisis with a view to the cleansing and disinfection of rooms.

[1] Dublin Jour. Med. Sci., Sept. 1. 1902. [2] Brit. Med. Jour., Sept. 13, 1902. [3] Sanit. Rec., Oct. 16, 1902. [4] Lancet, Nov. 29, 1902. [5] Sanit. Rec., Dec. 11, 1902. [6] Brit. Med. Jour., Dec. 20, 1902. [7] Sanit. Rec., Jan. 15, 1903. [8] Brit. Med. Jour., Jan. 24, 1903.

